# The Prognostic Value of Lymph Nodes Dissection Number on Survival of Patients with Lymph Node-Negative Gastric Cancer

**DOI:** 10.1155/2014/603194

**Published:** 2014-04-24

**Authors:** Wu Song, Yujie Yuan, Liang Wang, Weiling He, Xinhua Zhang, Chuangqi Chen, Changhua Zhang, Shirong Cai, Yulong He

**Affiliations:** Department of Gastrointestinal and Pancreatic Surgery, The First Affiliated Hospital of Sun Yat-sen University, 58 2nd Zhongshan Road, Guangzhou 510080, China

## Abstract

*Objective.* The study was designed to explore the prognostic value of examined lymph node (LN) number on survival of gastric cancer patients without LN metastasis. *Methods.* Between August 1995 and January 2011, 300 patients who underwent gastrectomy with D2 lymphadenectomy for LN-negative gastric cancer were reviewed. Patients were assigned to various groups according to LN dissection number or tumor invasion depth. Some clinical outcomes, such as overall survival, operation time, length of stay, and postoperative complications, were compared among all groups. *Results.* The overall survival time of LN-negative GC patients was 50.2 ± 30.5 months. Multivariate analysis indicated that LN dissection number (*P* < 0.001) and tumor invasion depth (*P* < 0.001) were independent prognostic factors of survival. The number of examined LNs was positively correlated with survival time (*P* < 0.05) in patients with same tumor invasion depth but not correlated with T1 stage or examined LNs >30. Besides, it was not correlated with operation time, transfusion volume, length of postoperative stay, or postoperative complication incidence (*P* > 0.05). *Conclusions.* The number of examined lymph nodes is an independent prognostic factor of survival for patients with lymph node-negative gastric cancer. Sufficient dissection of lymph nodes is recommended during surgery for such population.

## 1. Introduction

Gastric cancer (GC) is one of the most prevalent death-related cancers all over the world. Surgical resection combined with lymphadenectomy is a pivotal treatment for GC patients. From 1997 to 2002, the International Union Against Cancer (UICC) and the American Joint Committee on Cancer (AJCC) have established the lymph node (LN) classification of GC, which is mainly based on the number of metastatic lymph nodes (LNs) [1, 2]. It is required that at least 15 LNs must be dissected to achieve an accurate evaluation of N classification [3]. However, with this classification, the deviation of pathological TNM stage from clinical TNM stage still occurred in 10–15% of GC cases.

According to the 7th edition of UICC/AJCC TNM system, regardless of total number of excised LNs, N0 classification is defined as the negative LNs metastasis [1]. The system also indicates that the N classification actually depends on the number of positive LNs, rather than the number of examined LNs [4]. However, several studies have suggested that the total number of excised LNs is potentially related to the stage and prognosis of GC [5, 6].

To obtain a reliable assessment of N classification for GC patients, the UICC/AJCC recommends that at least 15 LNs should be dissected during surgery, irrespective of LN location [7, 8]. Although previous findings indicate that D2 lymphadenectomy with at least 15 LNs excised could provide a reliable prognosis for curable gastric cancer, it remains unclear whether the number of dissected LNs is correlated with clinical outcomes of lymph node-negative GC [9]. Hence, this study was designed to explore the relationship between the total number of examined LNs during surgery and the prognosis of GC without LN metastasis.

## 2. Patients and Methods

### 2.1. Patients

From August 1995 to January 2011, a total of 300 patients who had curable gastric cancer and underwent radical gastrectomy at our single center were retrospectively reviewed. Inclusion criteria were listed as follows. (1) Primary adenocarcinoma was confirmed histopathologically. (2) No lymph node or distant metastasis (any T stage, N0, and M0) was reported in final pathological diagnosis. (3) No history of gastrectomy or other malignancies was detected via reviewing medical records. Patients who suffered from advanced gastric cancer would receive additional chemotherapy prior to surgery, with alkylating drugs and fluorouracil as main chemotherapeutic agents. The clinicopathological characteristics of all patients were collected from our medical database. The protocol of current study was approved by the Research Ethics Committee of Sun Yat-sen University, with informed consent obtained from all patients or their legal representatives.

### 2.2. Methods

#### 2.2.1. Surgical Treatment

All patients who underwent radical gastrectomy plus D2 lymphadenectomy were included in final analysis. A curative tumor resection was defined as macroscopic removal of all visible tumors and metastatic LNs, with pathologically negative resection margins of 6–8 cm (R0 resection) achieved. Subtotal (proximal or distal) or total gastrectomy was selected according to the individual condition during surgery. Billroth I reconstruction was performed after a proximal gastrectomy, while Billroth II fashion was applied after a distal gastrectomy. Roux-en-Y anastomosis between esophagus and small intestine was applied after a total gastrectomy.

Primary tumors were resected en bloc with D2 lymphadenectomy according to the Japanese Gastric Cancer Association guidelines. D2 lymphadenectomy involves the removal of all LNs in N1 and N2 stations. The perigastric LNs near lesser curvature (stations 1, 3, and 5) and greater curvature (stations 2, 4, and 6) were grouped together as N1 station. LNs near left gastric artery (station 7), common hepatic artery (station 8), celiac artery (station 9), and splenic artery (stations 10 and 11) were grouped together as N2 station.

If the LNs around spleen or splenic vessels were invaded by tumors, spleen would be excised, with an extra-vessel sheath lymphadenectomy performed meanwhile. All LNs were harvested immediately from the resected specimen by experienced surgeons, as the standard procedure [10]. All harvested LNs were evaluated by two experienced pathologists, and the accurate number of dissected and positive LNs was reported in postoperative day 5.

According to the total number of dissected LNs, all patients can be artificially categorized into five groups: 1–15 LNs (*n* = 74), 16–20 LNs (*n* = 53), 21–25 LNs (*n* = 45), 26–30 LNs (*n* = 36), and over 30 LNs (*n* = 92) groups. Besides, these patients can be divided into T1 (*n* = 90), T2 (*n* = 55), T3 (*n* = 117), and T4 (*n* = 38) groups according to the T stage classification.

All patients had routinely been followed up after operation every 3 months for the first year, every 6 months for the second year, and twice a year thereafter. The routine examination during followup included a physical examination, blood chemistry, CXR, ultrasound of liver and abdomen, and bone scan the tumor marker levels. The follow-up period ranged from the first day of therapy until death or the last visit. The last following time in this study was January 2012. The survival time ranged from the first diagnosis of gastric cancer until the last contact after surgery, the date of death, or the date that the survival information was collected. In sum, nine patients were lost in the followup and the follow-up rate is 96%.

### 2.3. Statistical Analyses

Data were expressed as mean ± SD if not indicating otherwise. The Kaplan-Meier method was used to investigate overall survival rate and potential prognostic factors. Factors associated with clinical outcomes were assessed by univariate analysis for quantitative variables and *χ* ^2^ test for qualitative variables. Cox proportional hazards model was utilized in multivariate analysis. Spearman correlation analysis was used to evaluate the correlation between the number of LNs and survival time. The stepwise logistic regression was performed to evaluate the correlation between the number of dissected LNs and the incidence of postoperative complication. A *P* < 0.05 was considered statistically significant.

## 3. Results

### 3.1. Clinical and Pathological Data

A total of 300 patients (M : F, 16 : 1) were included in the final analysis, with average age of 56.3 (range: 26–81) years. Among this cohort, 7786 pieces of LNs were harvested during surgery (mean 26, range 10–91). The characteristics of enrolled subjects are shown in Table 1. There were no significant differences in age, gender, and tumor size or stage among the five different categorized groups. However, tumor location and depth of invasion had significant differences (*P* < 0.05).

### 3.2. Correlation between Dissected LNs and Clinical Outcomes

The clinical outcomes of all patients after surgery are shown in Table 2. There was no significant correlation between number of dissected LNs with time of operation (*P* = 0.621), volume of transfusion (*P* = 0.571), length of stay in hospital (*P* = 0.634), and incidence of postoperative complication (*P* = 0.228).

### 3.3. Univariate and Multivariate Survival Analyses

The average overall survival time of GC patients was 50.2 ± 30.5 months. By Cox proportional hazard analysis, cancer-specific survival was significantly correlated with number of dissected LNs (*P* < 0.001) and depth of tumor invasion (*P* = 0.001), but not related to age, gender, tumor size, tumor location, histological type, or grading (Table 3). By further multivariate regression, the two factors were considered as independent factors of survival for GC patients without LN metastasis.

### 3.4. Correlation between Examined LNs Number and Postoperative Complication

During the follow-up period, 12 patients (4%) suffered from postoperative complications, with one case for pancreatitis, three for anastomotic fistula, five for intra-abdominal abscess, two for bowel obstruction, and one for intra-abdominal hemorrhage. According to linear regression analysis, no significant correlation was detected between examined LNs number and complication incidence (*P* > 0.05).

### 3.5. Relation between Examined LNs Number and Survival Time under Different T Category

To evaluate the relationship between survival time and dissected LNs number, subgroup analysis based on the T category was performed subsequently. For patients with T1 tumors, no significant correlation was found between the two factors. For patients beyond T1 stage, the number of dissected LNs was positively correlated to the overall survival time. However, this correlation was not observed when the number of examined LNs was more than 30 (Table 4).

## 4. Discussion

In the present study, the findings indicate that the GC-specific survival for patients without LN metastasis was significantly correlated with the depth of tumor invasion and the number of dissected LNs during surgery. By subgroup analysis, increased number of dissected LNs was found to be helpful in prolonging survival time in GC patients beyond T1 stage.

LN metastasis is one of the most crucial indicators of poor prognosis for patients with resectable gastric cancer. Furthermore, the location and total number of examined LNs are independent factors in predicting survival of patients with gastric cancer. Previous studies have shown that total number of examined LNs was associated with postoperative pathological grading and overall survival. Properly increasing the number of examined LNs during surgery could further improve the accuracy of pathological grading, which is helpful in prediction of survival for GC patients [2, 11, 12].

The UICC/AJCC TNM classification recommends that at least 15 LNs should be examined to accurately evaluate pathological N category of gastric cancer. Additionally, LN metastasis rate is recommended to be considered as a prognostic factor of gastric cancer [13]. However, there is no consensus on the optimal number of examined LNs for GC patients with various T stages. Recently, a study indicated that at least 16 negative LNs had a strong correlation with the best disease-specific survival rate in GC patients beyond T1 stage [14].

It has been confirmed that LN-negative GC has similar clinicopathological features to early GC [15]. It was reported that the 5-year survival rate of LN-negative GC after curative gastrectomy plus D2 lymphadenectomy was 89.5% [16]. Besides, an insufficient amount of dissected LNs could lead to an unreliable pathological grading, and over 15 examined LNs were associated with improved prognosis of LN-negative GC compared with less than 15 dissected LNs [6]. Furthermore, an insufficient (<15) examined LNs number, even in early GC with T1 stage, was associated with a poor prognosis [17]. It has shown that increased number of examined LNs can reduce stage deviation and guide decision making for chemotherapy [2, 3, 8, 17]. On the other hand, LN micrometastasis may result in the inaccuracy of pathological N category [18, 19]. Therefore, increasing the number of examined LNs during surgery could reduce the chance of residual malignancy and improve the prognosis of gastric cancer.

In this study, patients who had gastric cancer without LN metastasis and underwent curative operation were retrospectively reviewed. However, due to individual pathophysiological conditions and various surgeon's preferences, there was a wide difference in dissected LNs number among all patients [20, 21]. As previously reported, older age and immune compromise were factors that might lead to an insufficient number of dissected LN [21, 22]. In our study, our results indicated that total number of dissected LNs and tumor invasion depth were independent prognostic factors of LN-negative GC. Actually, only the number of harvested LNs can be controlled by surgeons during operation. Hence, under current surgical skills and diagnostic techniques, it must be essential for surgeon to increase the total number of examined LNs when performing D2 lymphadenectomy.

The prognostic predictive value of total LN dissected number in survival of LN-negative GC had been reported, but no common consensus has been reached yet. Actually, the core problem focused on determination of optimal number of examined LNs during operation. Bouvier's study suggested that a reliable staging for LN-negative GC required at least 10 dissected LNs [23]. However, in his study, patients included for analysis mainly underwent D1 lymphadenectomy rather than D2 procedure as our study. It has been a controversial issue whether D1 or D2 operation should be considered as the standard procedure for advanced gastric cancer [24, 25]. Biffi and other colleagues suggested that at least 15 LNs should be dissected for LN-negative GC [6]. Baiocchi et al. reported that the total number of dissected LNs was related to the prognosis of LN-negative GC, with the highest survival rate achieved when at least 25 LNs harvested [26]. In our study, our results were in accordance with those of Baiocchi et al.

It has been proven that the total number of examined LNs is significantly related to the prognosis of patients with advanced gastric cancer [5]. In our study, we performed a stratified analysis according to T stage and found that the increased number of examined LNs was in proportion to the increased survival time for LN-negative advanced gastric cancer beyond T1 stage. However, this trend was not observed when the number of examined LNs exceeded 30.

In addition, the total number of examined LNs was also significantly related to the prognosis of early gastric cancer [17]. However, our study did not find a similar result. It has been reported that the LN metastasis rate in GC patient with T1 stage was 15–20% [27]. For such populations, there is no consensus on the recognized number of LNs examined during operation. Considering the difficulty of preoperative T staging, we still recommend that at least 15 LNs should be examined for early stage gastric cancer.

Several studies have suggested that postoperative complications were not correlated with the number of dissected LNs during surgery [28–30]. Those complications, such as anastomotic fistula, hemorrhage, and intestinal obstruction, were possibly related to surgeon's skills and perioperative management, rather than the range of LN dissection. In current study, the incidence of postoperative complications was not related to LNs dissected number, operation time, transfusion volume, or postoperative hospital stay. Therefore, properly increasing the number of dissected LNs during surgery would not increase the risk of postoperative complications for LN-negative GC.

In conclusion, our results indicate that 26–30 of dissected LNs from resected tumors during standard gastrectomy with D2 lymphadenectomy could increase overall survival of LN-negative GC. Properly increasing the number of harvested LNs during surgery is a safe procedure and would not increase the incidence of postoperative complications.

## Figures and Tables

**Table 1:** Overall survival univariate analysis for predictive factors of survival.

Variables	Number, *n* (%)	Survival, Mo	*χ* ^2^ value	*P* value
Gender			0.912	0.364
Male	205 (68.3%)	51.2 ± 27.3		
Female	95 (31.7%)	48.5 ± 26.7		
Age (years)			2.562	0.195
<60	172 (57.3%)	45.5 ± 24.6		
≥60	128 (42.7%)	50.7 ± 30.1		
Tumor Size (cm)			0.352	0.624
<4	185 (61.7%)	50.4 ± 27.2		
≥4	115 (38.3%)	46.5 ± 23.4		
Tumor Location			4.256	0.405
Upper third	42 (14.0%)	42.2 ± 27.7		
Middle third	52 (17.3%)	46.4 ± 27.2		
Lower third	206 (68.7%)	48.8 ± 32.7		
T Category			41.012	<0.001
T1	90 (30.0%)	46.1 ± 29.1		
T2	55 (18.3%)	48.9 ± 31.2		
T3	117 (39.0%)	53.0 ± 31.0		
T4	38 (12.7%)	55.0 ± 31.4		
Histopathological type			15.375	0.079
Adenocarcinoma	249 (83.0%)	41.3 ± 25.5		
Mucinous adenocarcinoma	21 (7.0%)	44.3 ± 18.4		
Signet ring cell carcinoma	30 (10.0%)	49.5 ± 14.5		
Histopathological grading			25.654	0.125
Well/medicate differentiated	199 (66.3%)	53.2 ± 24.7		
Poor/undifferentiated	101 (33.7%)	46.2 ± 26.7		
Examined Lymph nodes, *n*			46.23	<0.001
1–15	74 (24.7%)	43.7 ± 28.0		
16–20	53 (17.7%)	48.4 ± 25.5		
21–25	45 (15.0%)	52.7 ± 32.8		
26–30	36 (12.0%)	62.0 ± 31.3		
≥30	92 (30.6%)	49.7 ± 31.8		

Data in survival present with median ± SD. Chi-square test was used to compare the categorical variables. *P* < 0.05 considered statistically significant.

**Table 2:** Correlation between number of examined lymph nodes and clinical outcomes.

Variables	Overall	Number of examined lymph nodes					*X* ^2^(*F*) value	*P* value
		1–15	16–20	21–25	26–30	>30		
Hospital stay, day	15.0 ± 6.5	12.0 ± 5.5	13.5 ± 4.8	14.1 ± 6.5	16.1 ± 6.2	15.5 ± 4.8	0.431	0.634
Transfusion volume, ML	207.4 ± 400.4	188.3 ± 200.4	166.0 ± 66.9	120 ± 253.6	213.9 ± 398.7	248 ± 453	0.536	0.571
Operation time, min	266.3 ± 68.5	201.3 ± 56.4	266.3 ± 166.0	274.9 ± 57.5	273.3 ± 72.2	265 ± 72	0.576	0.621
Postop. Complication, *n* (%)								
No	288 (96.0%)	72 (97.3%)	51 (96.2%)	43 (95.6%)	34 (94.4%)	88 (95.7%)	3.423	0.228
Yes	12 (4.0%)	2 (2.7%)	2 (3.8%)	2 (4.4%)	2 (5.6%)	4 (4.3%)		

Data present as mean ± SD without specific statement. Postop: postoperative; chi-square test was performed to compare differences.

**Table 3:** Prognostic factors retained in multivariate analysis.

Factors	*P* value	Relative risk	95% CI
Invasion depth	<0.001	1.704	1.418–2.048
No. of examined lymph nodes	<0.001	1.616	1.468–1.779

Cox proportional hazard analysis was used to investigate the potential prognostic factors of survival in GC patients without lymph node metastasis.

**Table 4:** A stratified analysis between examined LNs and survival rate in all patients.

	*n*	1–15		16–20		21–25		26–30		>30		*P*
		*n*	ST	*n*	ST	*n*	ST	*n*	ST	*n*	ST	
T1	90	21	47.3 ± 28.9	17	49.8 ± 26.2	12	53.8 ± 18.8	14	54.1 ± 32.8	26	55.3 ± 32.3	0.06
T2	55	10	43.5 ± 29.3	13	44.1 ± 24.8	8	47.3 ± 47.1	6	55.0 ± 34.4	18	47.1 ± 25.2	0.002
T3	118	31	47.5 ± 27.4	15	51.8 ± 26.3	18	57.8 ± 35.4	14	71.0 ± 22.8	42	50.0 ± 33.5	0.000
T4	37	12	40.1 ± 41.0	8	48.2 + 14.1	9	50.2 ± 23.3	2	71.1 ± 35.5	6	46.8 ± 36.3	0.000

Sum	300	74	43.7 ± 28.0	53	48.4 ± 25.5	45	52.7 ± 32.8	36	62.0 ± 31.3	92	49.7 ± 31.8	0.000

ST: survival time (month for unit); *P* < 0.05 indicated a statistical significance by one-way ANOVA test.
